# The Knowledge of the Role of Papillomavirus—Related Head and Neck Pathologies among General Practitioners, Otolaryngologists and Trainees. A Survey-Based Study

**DOI:** 10.1371/journal.pone.0141003

**Published:** 2015-10-26

**Authors:** Joanna Jackowska, Anna Bartochowska, Michał Karlik, Mateusz Wichtowski, Maciej Tokarski, Małgorzata Wierzbicka

**Affiliations:** 1 Department of Otolaryngology, Head and Neck Surgery, University of Medical Sciences, Poznan, Poland; 2 Department of Phoniatrics and Audiology, University of Medical Sciences, Poznan, Poland; 3 Oncological Surgery Ward I, Greater Poland Cancer Center, Poznan, Poland; Istituto Nazionale Tumori, ITALY

## Abstract

**Objectives:**

The aim of the survey was to introduce knowledge of HPV's role in head and neck pathologies to general physicians (GPs), otorhinolaryngologists (ENTs) and newly graduated doctors, as well as to promote HPV-related diseases prevention.

**Study Design:**

Cross-sectional study.

**Methods:**

Self-designed questionnaire was sent to 2100 doctors. A total of 404 doctors, including 144 ENTs, 192 GPs and 68 trainees, responded.

**Results:**

The majority of ENTs (86.8%) had contact with recurrent respiratory papillomatosis (RRP) and oropharyngeal cancers (OPCs) patients; in contrast, the majority of GPs (55.7%) did not (p = 0.00). The knowledge of HPV aetiology of cervical cancer versus OPCs and RRP was statistically higher. 7% of ENTs, 20% of GPs and 10% of trainees had not heard about HPV in oropharyngeal diseases. Women had greater knowledge than men. Both in the group of GPs and ENTs, 100% of respondents had heard about the impact of vaccination on the reduction of cervical cancer incidence. Only 39.11% of respondents had heard about the possibility of using vaccination against HPV in RRP—ENT doctors significantly more often than GPs and trainees (p = 0.00). Only 28.96% of physicians had heard about the potential value of HPV vaccination in preventing OPCs, including 44.44% of ENT doctors, 23.44% of GPs and 11.76% of trainees (p = 0.00). The doctors from district hospitals showed lower level of knowledge compared with clinicians (p = 0.04).

**Conclusions:**

The different levels of knowledge and awareness of HPV issues highlight the need for targeted awareness strategies in Poland with implementation of HPV testing and vaccination. The information should be accessible especially to those with lower education levels: ENTs from small, provincial wards, GPs from cities of < 200 000 inhabitants and older physicians. The incorporation of HPV issues into the studies curriculum would be fruitful in terms of improving the knowledge of trainees.

## Introduction

Human papillomavirus (HPV) is a common sexually transmitted infection, and a well-documented agent in the development of cervical, vulvar, anal, penile and head and neck carcinomas. The high-risk HPV types 16 and 18 are implicated in over 70% of cervical cancer cases. The presence of HPV in oropharyngeal cancers (OPCs) is slightly lower: 60% in the USA, 40% in Europe (EUROCARE project) and 33% in other parts of the world, but it was documented that until 2000 the share of HPV infections was 41%, until 2004–72% and after 2004 there was an increase of up to 96% [1]. The low-risk HPV types 6 and 11 are responsible for vaginal and genital warts and the onset of recurrent respiratory papillomatosis (RRP) [2].

Testing for the high-risk types of HPV has been an established method in the cervical screening context around the world. The European Guidelines for Quality Assurance in Cervical Cancer Screening created the principles of organized population-based screening [3]. However, by July 2012, organized cervical screening programmes were available in only seven out of the 28 countries in the region, while the remaining 21 countries still have only opportunistic screening, mainly with insufficient funding and infrastructure, low population coverage and moderate to poor quality [4–5]. At present, only Slovenia has an effective, nationally organized cervical cancer screening programme with coverage of over 70% (2004–2008) [6].

In contrast to cervical cancer in the region of Central and Eastern Europe and Central Asia (CEECA), there is scarce data concerning the evaluation of the incidence and mortality from other HPV-related cancers and diseases [7]. Head and neck cancers are growing in a number of malignancies. The knowledge of these tumours has not been tested in European populations. American self-reported and objective measures indicate that few adults know much about the risk factors, such as tobacco use and HPV infection or common symptoms [8]. Patients sought information about HPV on the Internet but it was not easily navigable and thus physicians were a trusted source of information regarding HPV [9]. Strategies which are aimed at improving public awareness and decreasing the OPCs burden have to be included in physicians’ education which is the key to success.

The overall knowledge of HPV infections with special regard to the head and neck has never been examined in the influential milieu of Polish physicians. The aim of this survey was to introduce knowledge of HPV’s role in head and neck pathologies to general physicians (GPs), otorhinolaryngologists (ENTs) and newly graduated doctors. One purpose of the study was also to determine which demographic factors influenced this knowledge. The second aim was to test the hypothesis that the need for vaccination should be higher among people who have heard of HPV.

## Materials and Methods

**Fig 1:**
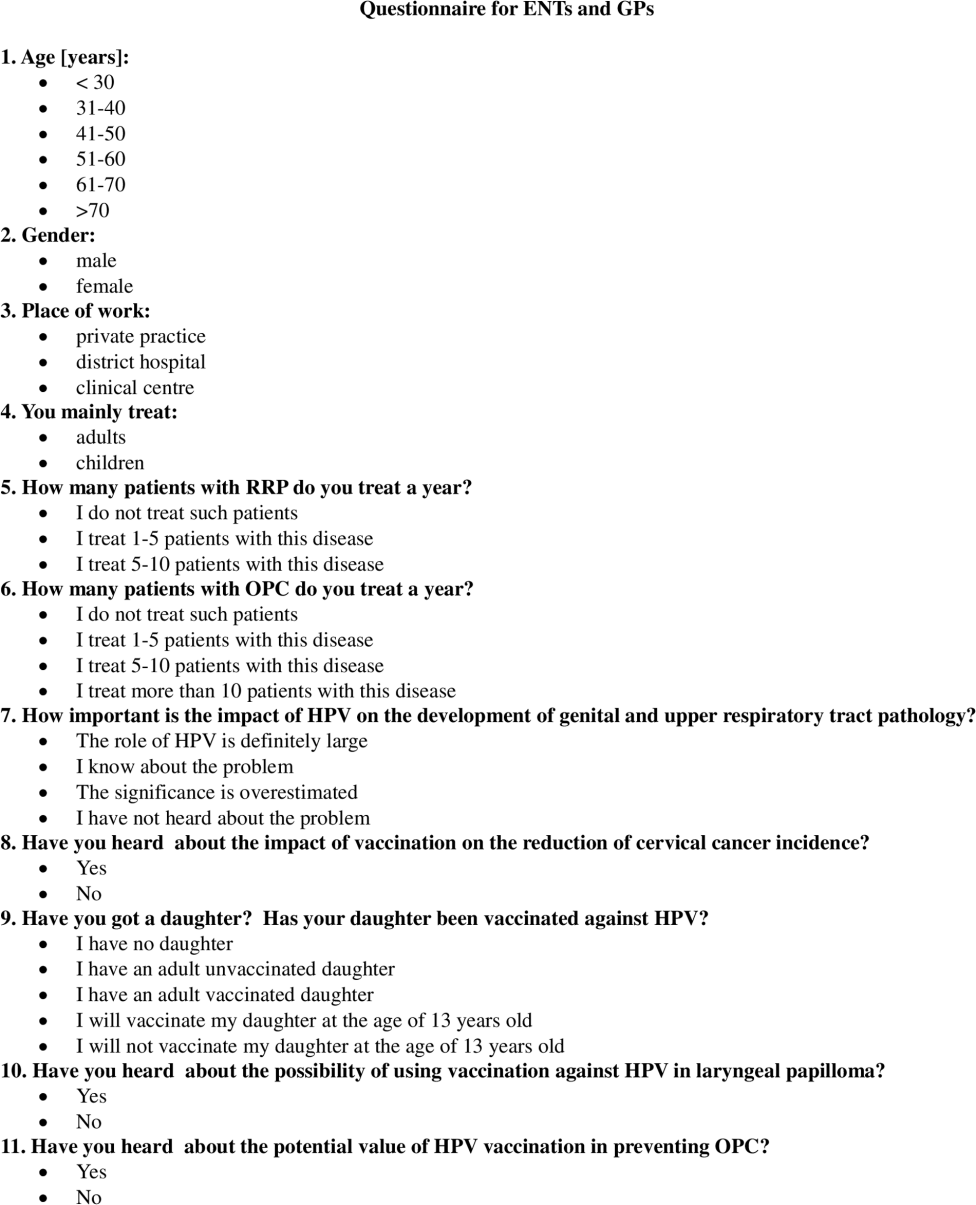
Questionnaire for ENTs and GPs.

The survey was conducted among otorhinolaryngologists (ear, nose and throat specialists—ENTs), general practitioners—GPs and trainees. For ENT doctors, GPs and trainees a questionnaire was constructed based on eleven slightly different questions (Fig 1). The survey was sent to 2100 doctors. A total of 404 doctors responded to the survey, including 144 ENTs, 192 GPs and 68 trainees. Each participant provided written consent to participate in the study which was accepted by the Bioethics Commission of Karol Marcinkowski Poznan University of Medical Sciences. The characteristics of the respondent group included the analysis of age, gender and place of work. The Chi-square test was used to compare the relationship between variables.

In the group of 144 ENT doctors the following age categories were analyzed: below 30 years old (n = 6), 31–40 years old (n = 26), 41–50 years old (n = 46), 51–60 years old (n = 51) and 15 respondents over 60 years old; 62 men and 82 women. The analysis of the workplace included: 48, 40 and 56 ENT doctors from clinical centres, district hospitals and private practices, respectively. 127 specialists treated adult patients and 17 treated children.

192 responses were obtained from family doctors, including 134 from women and 58 from men; the age group 41–50 years old was the most numerous (40%). Our respondents in a similar percentage (30%, 36%, 32%) came from population areas of up to 10 thousand, 10–200 thousand, and more than 200 thousand residents; 81% of them treated mostly adults.

The study analyzed the responses from 68 doctors who had just completed their studies and had not yet taken up a post. They constituted a homogeneous age group of people living in a city of > 200 thousand residents. The group of trainees included 44 women and 24 men.

Due to the fact that the studied traits are not quantitative (we cannot give values) but qualitative, the analysis of differences between the groups of ENTs, GPs and trainees can be carried out only through the study of relationships rather than differences.

## Results

### How many patients with RRP / OPC do you treat a year?

Two questions in the survey for ENTs and GPs related to the contact/treatment/care of patients with head and neck pathology of HPV aetiology: oropharyngeal cancer patients (OPCs: tonsils, base of tongue, lateral wall of the pharynx) and with recurrent respiratory papillomatosis (RRP). The following categories were examined: I do not treat such patients; I treat 1–5 patients with this disease; I treat 5–10 patients with this disease; the group with OPCs, which is a much more widespread disease than RRP, additionally included the category: I treat more than 10 patients with this disease. In the group of surveyed ENTs 99/144 treated patients from these groups. 38 ENT doctors treated 1–5 patients with RRP and 61 treated more than 5 such patients. 125 ENTs took care of OPCs patients, 48 treated 1–5 patients a year, 19 treated 5–10 patients a year and 58 treated more than 10 patients a year. The vast majority of GPs (77%) did not take care of patients with RRP but almost 45% had patients with OPCs. Differences between the two professional groups in terms of contact with patients with RRP and OPCs were significant (p = 0.00, p = 0.00); ENT doctors took care of these patients significantly more often (Table 1).

**Table 1:** Comparison of the number of patients with RRP and OPCs under the care of ENT doctors and GPs.

Disease category	RRP		OPCs
Group of doctors	ENTs (144)	GPs (192)	ENTs (144)
I have no such patients	45 (31.25%)	147 (76.56%)	19 (13.19%)
I treat 1–5 patients with this disease	61 (42.36%)	42 (21.88%)	48 (33.33%)
I treat 5–10 patients with this disease	38 (26.39%)	3 (1.56%)	19 (13.19%)
I treat more than 10 patients with this disease	I have no such number of RRP or OPCs patients	I have no such number of RRP or OPCs patients	58 (40.28%)

### How important is the impact of HPV on the development of genital and upper respiratory tract pathology?

Respondents from the three groups: GPs, ENT doctors and trainees answered how important, in their opinion, the impact of HPV was on the development of genital and upper respiratory tract pathology. Knowledge of the importance of HPV to the carcinogenesis of cervical carcinoma was widespread; awareness of HPV’s role was 100%, 85% and 98.5% of respondents in the group of ENTs, GPs and trainees, respectively. The importance of HPV to the development of upper respiratory tract pathology was not as common. It was tested in the following categories: the role of HPV is definitely large; I know about the problem; the significance is overestimated; I have not heard about the problem (Table 2). A relationship between the groups of ENTs and GPs in terms of the assessment of the importance of HPV’s role in upper respiratory tract pathology was in the category "large" and the rest of the categories were statistically significant (p = 0.00), similarly when considering the total category of "large" + "I know the problem," and "overestimated " + "not heard" (p = 0.00). In contrast, a relationship between ENTs and trainees in terms of the assessment of the significance of HPV’s role in upper respiratory tract pathology was in the category "large", and the rest were not statistically significant (p = 0.66), just as in the total consideration of the category "large" + "I know the problem," and "overestimated " + "not heard" (p = 0.64). The assessment of HPV’s importance in the development of upper respiratory tract pathology differed in the group of ENT doctors depending on whether they treated adults or children; high importance of HPV was attributed to 52% of otolaryngologists treating adults but only 6% of paediatric ENTs (p = 0.05).

**Table 2:** Summary of the opinions of doctors of various professions regarding the importance of HPV infection to the development of pathology of the cervix, pharynx and larynx.

Category	ENTs (N = 144)		GPs (N = 192)		Trainees (N = 68)	
	Pharynx/larynx	Cervix	Pharynx/larynx	Cervix	Pharynx/larynx	Cervix
Large	85 (59%)	100%	55 (28.6%)	164 (85.4%)	38 (55.9%)	67 (98.5%)
I know the problem	47 (32.6%)	-	86 (44.8%)	22 (11.5%)	23 (33.8%)	1 (1.4%)
Overestimated	11 (7.6%)	-	13 (6.8%)	5 (2.6%)	-	-
I have not heard about the problem	1 (0.7%)	-	38 (19.8%)	1 (0.5%)	7 (10.3%)	-

### Have you heard about the impact of vaccination on the reduction of cervical cancer incidence?

Both in the group of GPs and ENTs, 100% of respondents had heard about the impact of vaccination on the reduction of cervical cancer incidence.

### Have you got a daughter? Has your daughter been vaccinated against HPV?

In the group of ENT doctors and GPs, 47% had a daughter. To the question: Has your daughter been vaccinated against HPV? respondents gave the following answers: ENT doctors: I have an adult, unvaccinated daughter—22,9%; she has been vaccinated—17,4%; I will vaccinate my daughter—15,9%; I will not vaccinate my daughter—3,5%; whereas in the group of GPs: 20,8% had an adult daughter vaccinated against HPV, 12,5% had an unvaccinated daughter, 13% planned to vaccinate a daughter, and 1,6% did not plan to vaccinate (Table 3).

**Table 3:** Comparison of the group of ENTs and GPs in terms of using vaccination against HPV in their daughters.

Category	ENTs (144)	GPs (192)
I have no daughter	58 (40.3%)	100 (52.1%)
Adult, vaccinated	25 (17.4%)	40 (20.8%)
Adult, unvaccinated	33 (22.9%)	24 (12.5%)
I will vaccinate at the age of 13 years old	23 (15.9%)	25 (13%)
I will not vaccinate at the age of 13 years old	5 (3.5%)	3 (1.6%)

### Have you heard about the possibility of using vaccination against HPV in laryngeal papilloma?

A total of 158/404 (39.11%) physicians had heard about the possibility of using vaccination against HPV in laryngeal papilloma: 88/144 (61.11%) ENT doctors, 59/192 GPs (30.73%) and 11/68 (16.18%) trainees. Knowledge of the problem differed significantly between the groups of ENT doctors and GPs (p = 0.00) and the group of ENTs and trainees (p = 0.00).

### Have you heard about the potential value of HPV vaccination in preventing OPC?

A total of 117/404 (28.96%) physicians had heard about the potential value of HPV vaccination in preventing OPC, including 64/144 (44.44%) ENT doctors, 45/192 (23.44%) GPs and 8/68 (11.76%) trainees. Knowledge of the problem differed significantly between the groups of ENTs and GPs (p = 0.00) and the group of ENTs and trainees (p = 0.00).

### ENTs

The analysis of the data characterizing the group of ENTs and the data describing the state of their knowledge of HPV indicates that the age of respondents did not matter in terms of the knowledge about HPV or the possibility of using vaccination against HPV in laryngeal papilloma or precancerous conditions of the oral cavity and pharynx. The age of respondents, however, had a significant influence on the decision whether to vaccinate daughters: the younger the age of respondents, the greater their willingness to vaccinate previously unvaccinated adolescent girls (p = 0.00006).

In the group of ENT doctors gender was important in assessing the role of HPV in the development of upper respiratory tract pathology: HPV was considered a crucial pathological factor by 20% of men and 39% of women (p = 0.009). This predilection, however, did not affect the state of knowledge about the role of vaccines in RRP or precancerous conditions of the oral cavity and pharynx or the decision to vaccinate daughters.

The workplace of ENT doctors was important in the assessment of the significance of HPV in the development of upper airway pathology (p = 0.00025): 28% of employees of clinical centres assessed the importance as very high, whereas only 11% of the staff of district hospitals and 18% of employees of private practices shared this view; the last two groups assessed the role of HPV as significant (11% and 18%). The place of work did not affect the attitude to vaccination, both in terms of using it in own daughters as well as the option for patients with RRP. As for the potential importance of vaccination to protect against the development of OPC, doctors working in clinical centres (19%) and private practices (16%) gave significantly higher percentages of affirmative responses compared to the staff of district hospitals (9%) (p = 0.04).

### GPs

The analysis of the data characterizing the group of GPs and the data describing the state of their knowledge of HPV showed that the age of respondents did not significantly affect the way they perceived the importance of HPV in the development of upper respiratory tract pathology, knowledge of the potential role of vaccination against HPV in laryngeal papilloma, and the prevention of OPCs. Age, however, was crucial in the decision whether to vaccinate daughters. The cohort of 41–50 year-olds was the most enthusiastic to take this action compared to older doctors (p = 0.00). The gender of the surveyed physicians did not affect any of the studied parameters.

There were no significant differences in terms of the workplace of GPs (size of the city) that would influence the assessment of the importance of HPV in pathology of the cervix, genitals and upper respiratory tract, the possibility of vaccination against HPV in laryngeal papilloma, and the role in OPC prevention. In contrast, a desire to vaccinate daughters was declared mostly by respondents of cities of over 200 thousand inhabitants (p = 0.02).

### Trainees

The analysis of the data characterising the group of trainees and the data describing the state of their knowledge showed that the gender of respondents did not affect the assessment of HPV’s significance in the development of pathology of the cervix, genitals and upper respiratory tract. 98% had heard about the impact of vaccination on the reduction of cancers of the cervix. However, when asked the questions: Have you been vaccinated? Has our partner been vaccinated?, as many as 82% of respondents answered that they had not: 15% of the surveyed women, and fewer than 3% of the partners of the surveyed men were vaccinated.

## Discussion

The increasing knowledge about HPV’s significance in head and neck pathology may help to minimize any negative psychological consequences associated with HPV testing and vaccination. Medical recommendations for cancer screening and prevention are connected with the compliance of the patient [8]. Thus, the aim of this survey was to introduce knowledge of HPV’s role in head neck pathologies to physicians in a Middle European country with a population of 38 million. By recruiting online we were able to directly compare samples across three subgroups of doctors, using a survey regarding HPV knowledge issues. Intentionally, the questionnaire was very short, with closed-questions and took two minutes to complete online. The sample was limited to two medical specialities: ENTs, and GPs and trainees, as the main target was to examine them on their knowledge of the most frequent non-anogenital HPV-related diseases, RRP and OPCs.

In contrast with the USA, where HPV vaccination has been presented through public health initiatives, with an overriding sense of community obligation to be vaccinated for whatever herd immunity may occur [10–11], in Poland it is still the subject of debate [12]. Healthy People 2020 aims for very high population vaccination rates [13] but recent surveys indicate that only a portion of the population is willing to consider HPV vaccination [14]. A few studies were dedicated to assist in developing intervention strategies for engaging different medical groups, for instance dentists, advanced practice registered nurses, and physician assistants, in discussing HPV vaccine with patients [15–16]. We have not found in the literature surveys conducted among physicians which concern knowledge and awareness of HPV aetiology in upper airway pathologies and its influence on the decision to be vaccinated against HPV infection. Raising awareness in Polish physicians could become increasingly important if HPV testing and vaccination are introduced into the routine diagnosis and management of cancers other than cervical HPV-related [17].

In the presented analysis 404 Polish physicians responded to the survey. Taking into consideration the everyday contact with RRP and OPCs patients, the majority of ENTs (125/144) have contact with these entities; in contrast, the majority of GPs (107/192) do not. The knowledge of HPV aetiology of cervical cancer versus OPCs and RRP was statistically higher. Around two thirds of those who had heard of HPV in ano-genital pathologies had also heard of HPV in oropharyngeal diseases. However, 7% of ENTs, 20% of GPs and 10% of trainees had not heard about the problem. The differences were significant and indicate that GPs should be a target for the intensification of education. Women had greater knowledge than men, which could have been expected, since HPV is commonly associated with cervical cancer, so it is women who are currently referred for HPV testing.

In the group of ENT doctors the workplace was important in the assessment of HPV’s significance in the development of upper respiratory tract pathology: 28% of employees of clinical centres assessed this significance as very high, whereas only 11% of the staff of district hospitals and 18% of employees of private practices shared this view. As for the potential importance of vaccination to protect against the development of OPCs, doctors working in clinical centres (19%) and private practices (16%) gave significantly higher percentages of affirmative responses compared to the staff of district hospitals (9%) (p = 0.04). In both categories doctors from district hospitals showed the lowest level of knowledge compared with clinicians. The high level of self-education in doctors running their own practices is noteworthy.

The high impact of medical recommendations for vaccination has been proven [18–19]. Of note, the recommendation for any one vaccine had a positive effect on the uptake of other vaccines [18]. HPV vaccination has failed to achieve the uptake comparable to other adolescent-specific vaccines. Among the most commonly cited reasons for receiving vaccines were recommendations from a health care provider and concerns for adverse effects. Lack of time to educate parents about the HPV vaccine and the cost of the vaccine were two other commonly reported barriers to offering the vaccine by providers. The authors conclude that the majority of providers serving the highest risk populations for HPV infection and HPV-related cancers do not routinely recommend the HPV vaccine to their patients [19]. This demonstrates where knowledge could be improved. Surprisingly, our study demonstrated, in the group of respondents who had a daughter at the HPV vaccination age, a disproportion between knowledge of HPV issues and the willingness to vaccinate. It is obvious that the lower knowledge of HPV-related pathologies may reflect the limited use of the HPV vaccines. But we have proven that the awareness of HPV in cervical cancer or other HPV-related cancers is not parallel with the knowledge of the benefits of the HPV vaccination. The knowledge of HPV’s importance in carcinogenesis was widespread in terms of cervical carcinoma: 100%, 85% and 98.5% of ENTs, GPs and trainees, respectively. 100% of respondents considered vaccinations to be important. Although the sample had heard about HPV and appreciated the vaccines, this did not translate into greater willingness to vaccinate daughters against HPV. A higher willingness to vaccinate daughters was affected by only two factors: younger age in the groups of ENTs and GPs and the place of work in places with > 200 000 inhabitants in the group of GPs.

There are some limitations of the study. Participants were recruited from online panels and so may not be representative of the whole population of doctors but they are probably the most open and questing group. To summarize, the different levels of knowledge and awareness of HPV issues highlight the need for targeted awareness strategies in Poland where we would like to implement HPV testing and vaccination. Knowledge and awareness could be improved through ensuring that information is accessible to those with lower education levels: ENTs from small, provincial wards, GPs from cities of < 200 000 inhabitants and older physicians. Thus, it may be helpful to encourage the discussion about HPV testing and vaccination in the media and to incorporate information about HPV and the implications of HPV test results in community outreach initiatives. The incorporation of HPV issues into the studies curriculum would be fruitful in terms of improving the knowledge of trainees.
